# Obesity Measures and Dietary Parameters as Predictors of Gut Microbiota Phyla in Healthy Individuals

**DOI:** 10.3390/nu12092695

**Published:** 2020-09-03

**Authors:** Katja Bezek, Ana Petelin, Jure Pražnikar, Esther Nova, Noemi Redondo, Ascensión Marcos, Zala Jenko Pražnikar

**Affiliations:** 1 Faculty of Health Sciences, University of Primorska, Polje 42, SI-6310 Izola, Slovenia; katja.bezek@upr.si (K.B.); ana.petelin@upr.si (A.P.); 2 Faculty of Mathematics, University of Primorska, Natural Sciences and Information Technologies, Glagoljaška 8, SI-6000 Koper, Slovenia; jure.praznikar@upr.si; 3 Immunonutrition Group, Department of Metabolism and Nutrition, Institute of Food Science, Technology and Nutrition (ICTAN), Spanish National Research Council (CSIC), Jose Antonio Novais, St.10., 28040 Madrid, Spain; enova@ictan.csic.es (E.N.); noemi_redondo@ictan.csic.es (N.R.); amarcos@ictan.csic.es (A.M.)

**Keywords:** gut microbiota, nutrition, obesity measures, lifestyle parameters, clustering, machine learning

## Abstract

The dynamics and diversity of human gut microbiota that can remarkably influence the wellbeing and health of the host are constantly changing through the host’s lifetime in response to various factors. The aim of the present study was to determine a set of parameters that could have a major impact on classifying subjects into a single cluster regarding gut bacteria composition. Therefore, a set of demographical, environmental, and clinical data of healthy adults aged 25–50 years (117 female and 83 men) was collected. Fecal microbiota composition was characterized using Illumina MiSeq 16S rRNA gene amplicon sequencing. Hierarchical clustering was performed to analyze the microbiota data set, and a supervised machine learning model (SVM; Support Vector Machines) was applied for classification. Seventy variables from collected data were included in machine learning analysis. The agglomerative clustering algorithm suggested the presence of four distinct community types of most abundant bacterial phyla. Each cluster harbored a statistically significant different proportion of bacterial phyla. Regarding prediction, the most important features classifying subjects into clusters were measures of obesity (waist to hip ratio, BMI, and visceral fat index), total body water, blood pressure, energy intake, total fat, olive oil intake, total fiber intake, and water intake. In conclusion, the SVM model was shown as a valuable tool to classify healthy individuals based on their gut microbiota composition.

## 1. Introduction

The human gut microbiota is a complex and dynamic community of bacteria, viruses, fungi, protozoa, and archaea, which coexist in symbiosis with the host [1]. Due to the relative abundance of the Bacteria domain and its role in health and disease of the host this part of gut microbiota has been widely studied. The density of bacterial community along the gastrointestinal (GI) tract varies and is the highest in the colon that harbors 10^12^ bacteria/gram of intestinal content [2]. A significant contribution of gut bacteria in the functionality of immune, GI, and nervous system has been observed, including (i) maturation and regulation of the immune system [3], (ii) intestinal mucosal barrier maintenance [4], (iii) protection against pathogens [5], and (iv) balancing energy metabolism and hormone levels [6]. Moreover, the disturbance in the gut microbiota homeostasis has been associated with gastrointestinal disorders [7], metabolic diseases [8], as well as with neurological disorders [9].

Although individually specific, the gut microbiota of a healthy adult is composed mainly of two dominant bacterial phyla Firmicutes and Bacteroides (90%) followed by other phyla, including Actinobacteria (mainly *Bifidobacterium*), Proteobacteria, Fusobacteria, and Verrucomicrobia in lower proportion [10]. While the key events of gut microbiota development are set already in early life [11], the microbial community can daily be influenced by a wide range of environmental and inter-individual variables, including diet, medications, and a wide range of anthropometric measurements [12]. The gut bacteria composition, diversity, and/or functional richness of a healthy individual have been shown before to be correlated with variables such as age and sex [12,13], obesity [14,15], diet (e.g., fiber intake, a diet rich in carbohydrates or proteins) [16,17]. Although diet and medications, specifically antibiotics, are recognized as the most important factors modulating the diversity and function of the gut microbiota, this explains only a small proportion of variations [12,18,19]. Interestingly, a negligible influence of host genetics on fecal microbiome composition was observed [20]. The last suggests that interventions on microbiota composition with the aim of beneficial health outcomes may be carried out across diverse genetic backgrounds [12].

However, it is still an open question which of the gut microbiota-associated variables could serve as predictors of phyla and genera abundance in healthy individuals. It is conceivable that the gut microbiota composition could then yield important information about the physiological state of the host. The classification from microbiome data has already been performed recognizing the support vector machines (SVM) as one of the most effective and accurate machine learning techniques [21]. The first aim of the present study was the clustering of healthy subjects according to the bacteria phyla abundance. Secondly, we aimed to determine which subject’s characteristics, such as age, gender, BMI (body mass index), macro- and micronutrient intakes, level of physical activity, alcohol consumption, daily sleeping hours, and others are of the highest importance in allocating healthy individuals into a single cluster, using SVM. The obtained data could provide us with a comprehensive check on microbiota-associated variables that simultaneously influence the allocation of healthy subjects into clusters.

## 2. Materials and Methods

### 2.1. Study Design

The present study was a part of a larger project on the effect of alcohol consumption on gut microbiota (ALMICROBHOL). This cross-sectional study has been approved by the Slovenian National Medical Ethics Committee (No. 53/03/2015) and was performed according to the Declaration of Helsinki (the latest amendment in Fortalesa, Brasil, 2013) at the Faculty of Health Sciences, University of Primorska, Izola, Slovenia, between October 2014 and December 2016. Prior to the study, all of the participants submitted a written informed consent.

The participants who met the inclusion criteria followed a two-visit schedule. In the first visit, participants completed questionnaires about physical activity, alcohol consumption and macro- and micronutrient intakes. Anthropometrical measurements, blood pressure, and questionnaires about overall health status and quality of life were performed in a second visit. All questionnaires were filled in by a trained investigator in personal interviews with the participant. In addition, all questionnaires were validated and translated into English and from English to the Slovenian language. For gut microbiota analysis, each participant brought a fresh stool sample that was collected in a sterile container at home the day before an in-person visit. The samples were immediately stored at −80 °C and collectively transported on dry ice to the Spanish Institute of Food Science, Technology and Nutrition (ICTAN, Madrid, Spain) for further analysis.

### 2.2. Study Subjects

For this cross-sectional study, 261 healthy individuals (Caucasian origin) from the general population were recruited by advertisement (internet forums, e-mail and newspaper advertisements). Only subjects who met the following inclusion criteria were eligible to participate: (a) aged 25–50 years; (b) body mass index (BMI) between 18.5 and 35 kg/m^2^; (c) healthy with no cardiovascular, endocrine, GI, or acute or chronic inflammatory diseases; (d) not taking nonsteroidal anti-inflammatory drugs or antibiotics 90 days prior to the study; and (e) reporting a stable weight within the last three months. Among 261 potential subjects, 61 were excluded from the study because of the missing questionnaire data or stool sample for microbiota analysis.

#### 2.2.1. Dietary Assessment

Study subjects first underwent baseline energy intake assessments, including a three-day accurate food recording and an assessment of a 30-day validated food frequency questionnaire. The last estimates the amount and consumption frequency of 209 food items including both foods, water intake and beverages over the past one-month period [22]. One week prior to stool sample collection the subjects were instructed to record and weigh their food intake for three consecutive days (two days during the week and one weekend). Where possible, subjects were asked to include food labels and recipes for mixed dishes and were encouraged to avoid any alterations to their normal diet. They were taught to weigh and record all food and beverages immediately before eating and to weigh and describe any leftovers. Dietary data (energy values and macro- and micronutrient content) were analyzed using a freely accessible online dietary assessment and planning tool, named Open Platform for Clinical Nutrition (http://www.opkp.si/en_GB/cms/vstopna-stran). Moreover, adherence to the Mediterranean diet was investigated according to the 14-item Mediterranean diet adherence score used in the PREDIMED study. Based on the gained score, subjects adherence to the Mediterranean diet was classified as low (0–7), moderate (8–10), or high (11–17) [23]. In addition, the specific consumption of alcoholic beverages was determined by an ad-hoc modified questionnaire of the SUN study [24].

#### 2.2.2. Body Composition Measurements

Subjects’ height, weight, waist and hip circumference, as well as blood pressure were measured using a standardized protocol. All measurements were performed by the same examiner between 7 a.m. and 9 a.m. The height and weight of participants were measured in light indoor clothing without shoes with a precision of 0.1 cm and 0.1 kg. The waist measurements were performed in a standing position halfway between the coastal edge and iliac crest, whereas for hips the greatest circumference around the buttocks as measured. BMI was calculated by the formula BMI = (weight in kg)/(height in m)^2^ and waist to hip ratios by the formula WHR = waist (cm)/hip (cm). Body composition (total percentage of body fat (% BF) and percentage of trunk fat (% TF) were assessed by bioelectrical impedance analysis (BIA) using a Tanita BC 418MA, followed by data analysis in software provided by the manufacturer (Tanita Corporation, Arlington Heights, IL). Although there is a correlation between % BF and % TF, visceral fat is better presented by % TF. In addition, the same analysis provided also the visceral fat rating data.

#### 2.2.3. Overall Health Status and Quality of Life

Overall health status, diagnosed diseases, symptoms, drug administration, and sleep quality were evaluated through the interview. Socio-economic status (academic background, incomes, economic supports, and family members) and daily activities (hours spent for personal hygiene, shopping, cooking, and cleaning and tidying the house) were assessed by the modified AFINOS study questionnaire [25]. Subjective self-perception of physical and emotional health and pain were determined by the Short Form-36 Health Survey questionnaire [26]. In addition, the physical activity of participating subjects was assessed using the Minnesota Leisure Time Physical Activity Questionnaire. EER was estimated according to Institute of Medicine of the National Academies, accessible online: https://www.nap.edu/read/10490/chapter/1.

#### 2.2.4. Gut Microbiota Composition

The gut microbiota composition was determined using the MiSeq Illumina system (Illumina, San Diego, CA, USA) as previously described [27]. Briefly, after the bacterial DNA extraction using an optimized protocol [28], the DNA was recovered with the commercial QIAamp DNA Stool Mini Kit (Qiagen N.V., Venlo, The Netherlands) following the manufacturer’s instructions and stored at −80 °C for further analysis. DNA concentration was measured by UV absorbance at 260 nm, and the DNA quality was also assessed by the 260/280 nm ratio using a Nanodrop ND-1000 spectrophotometer (Thermo Fisher Scientific, Wilmington, DE, USA). Subsequently, Picogreen analysis of double-stranded DNA was performed by a QuantiFluor ST fluorometer (Promega, Madison, WI, USA), and all samples were diluted to a final concentration of 0.5 ng/µL for the amplification of the V3–V4 variable regions of the 16S RNA gene. DNA amplicon integrity was checked by 1.5% agarose gel electrophoresis (Pronadisa, Madrid, Spain), and libraries were normalized and pooled. Sequencing was performed by the MiSeq Illumina system (Illumina, San Diego, CA, USA) using the V3 kit on a 2 × 270 paired-end runs.

The taxonomy classification was performed with the MiSeq Reporter software (v2.3, Illumina) in several steps, including demultiplexing and FASTq file generation, as described before [27]. Sequences were then clustered into operational taxonomic units (OTUs) with Classify Reads, a high-performance implementation of the Ribosomal Database Project (RDP) based on the Greengenes database, obtaining 26 phyla, 605 genera, and 1790 species (Classify Reads accuracy was 100%, 99.97%, and 98.65%, respectively) [29]. Taxa with relative abundance <0.002% of total reads and also those with a prevalence of <10 subjects were removed for the statistical analysis. For the present study, the data about relative abundance was obtained from the Illumina 16S Metagenomic Report of the gut microbiota from an individual stool sample, provided by the ICTAN (Madrid, Spain). Data with relative abundances of phyla and genera of 200 subjects that were included in the present study are presented as Supplementary Material (Table S1).

### 2.3. Data Analysis

The resulting data in the context of the survey questioners and gut microbiota analysis were entered into a database. Frequencies were compiled for all variables and are presented as percentages for nominal and as mean or median and standard deviation (SD) for numeric variables. Quantitative data analysis with the application of statistical software was performed in SPSS software version 23 (IBM, Armonk, NY, USA). An R package version 3.5 was used for pre-processing data and for performing hierarchy clustering using Ward’s linkage method. The caret package in R was used to build the Support Vector Machine (SVM) classifier. For the SVM classification model, the variable importance was calculated by computing the area under the Operating Characteristic (ROC) curve. The Variable importance function, which is a part of the caret package in R, automatically scales the importance scores to be between 0 and 100.

#### 2.3.1. Cluster Analysis of Gut Microbiota

Hierarchical clustering using Ward’s linkage of the correlation coefficient as a distance measure was performed to analyze the microbiota data set. For clustering, the data that met the next criteria were used: most abundant phyla with more than 50% of the data above detection limit. According to the criteria, data of five phyla (Firmicutes, Bacteroidetes, Proteobacteria, Actinobacteria, and Verrucomicrobia) of 200 healthy individuals were used. Before the analysis, the data set were scaled so that all variables have zero mean and unit variance.

Pearson correlation distance was used as a similarity measure (distance) between variables. The correlation distance (D) is defined as D = 1 − R, where R is the Pearson correlation coefficient. We choose correlation distance as a similarity measure because the main interest was to inspect the strength of the linear relationship between variables. The Ward’s linkage, a minimal increase of sum-of-squares, was used to calculate the distance between clusters. Hierarchical clustering results in a clustering structure consisting of nested partitions. In an agglomerative clustering algorithm, the clustering begins with singleton sets of each point. That is, each data point is its own cluster. At a single time step, the most similar cluster pairs are combined according to the chosen similarity measure, and this step is repeated until predetermined criteria are met.

#### 2.3.2. Predicting Microbiota Clusters Using Support Vector Machine

Support Vector Machine (SVM) is a supervised machine learning technique that is widely used for classification challenges. The SVM algorithm performs classification by constructing multidimensional planes that define decision boundaries. In this study, the Radial Basis Function (RBF) kernel was applied to discriminate four cluster classes. Seventy variables from data collected on demographics, anthropometrics, lifestyle, and socio-economic status were included to build the SVM model. The detailed description of variables is presented in the Table S2 of the Supplementary Materials. The variables were selected (1) to illustrate RBF performance when dealing with a high-dimensional biomedical problem combining continuous and categorical traits and (2) to uncover potentially unknown predictors of microbiota composition. Variable selection was also guided by biological plausibility and was limited to variables with less than 5% missing data. The SVM model was validated using a Leave-one-out cross-validation setup, which is a special case of cross-validation. Leave-one-out cross-method performs training on the whole data set minus single observation. The single observation, which is not a part of the training set, is used for the validation. Leave-one-out cross-validation is repeated k-times where k is the number of observations in the original sample. RBF has two main parameters: C controls the cost of miss-classification, and gamma determines the radius of influence of the support vectors. Thus, the systematic grid search in a combination of Leave-one-out cross-method was performed for the search of best C and gamma values. The values of C and gamma where the highest accuracy was achieved were selected as the optimal parameter set. The optimal parameters C and gamma were 7 and 0.03, respectively. The performance of the model was additionally estimated by a calculation of confusion matrix that shows in which way the model is confused when it makes predictions. The accuracy of the model was 0.70, where a 95% confidence interval was between 0.62 and 0.75. Furthermore, the balanced accuracy, which considers the imbalance between classes and is defined as the arithmetic mean of sensitivity (true positive rate) and specificity (false positive rate), was 0.783, 0.796, 0.791, and 0.712, for Cluster 1, Cluster 2, Cluster 3, and Cluster 4, respectively.

## 3. Results

### 3.1. Characteristics of the Study Subjects

A total of 200 healthy Caucasian adults (117 females and 83 males) were included in the present study of whom basic characteristics are provided in Table 1. The average age of the participants was 35.4 ± 7.0 years and most of them had a university degree (63.5%). The participants slept on average 7.7 ± 0.8 of nightly hours. Regular cigarette smoking was reported by 27% of them, and the average alcohol consumption was 14.5 ± 13.6 g/day. Regarding obesity parameters, the average BMI was 24.2 ± 3.5 kg/m^2^, WHR = waist (cm)/hip (cm) was 0.87 ± 0.07, and visceral fat index was 4.7 ± 2.9%. The mean Estimated Energy Requirements (EER) of participants was shown to be 3093 ± 776 kcal, and the estimated daily energy intake was 1892 ± 1000 kcal/day, followed by total fat intake of 32.07 ± 9.96%; 49.05 ± 10.18% of carbohydrates and 18.14 ± 6.48% of proteins. The average amount of consumed fiber was 19.37 ± 13.67 g/day. In the context of the Mediterranean diet, the adherence of most (59%) participants was moderate and for 11% high.

### 3.2. Microbiota Composition

**Figure 1:**
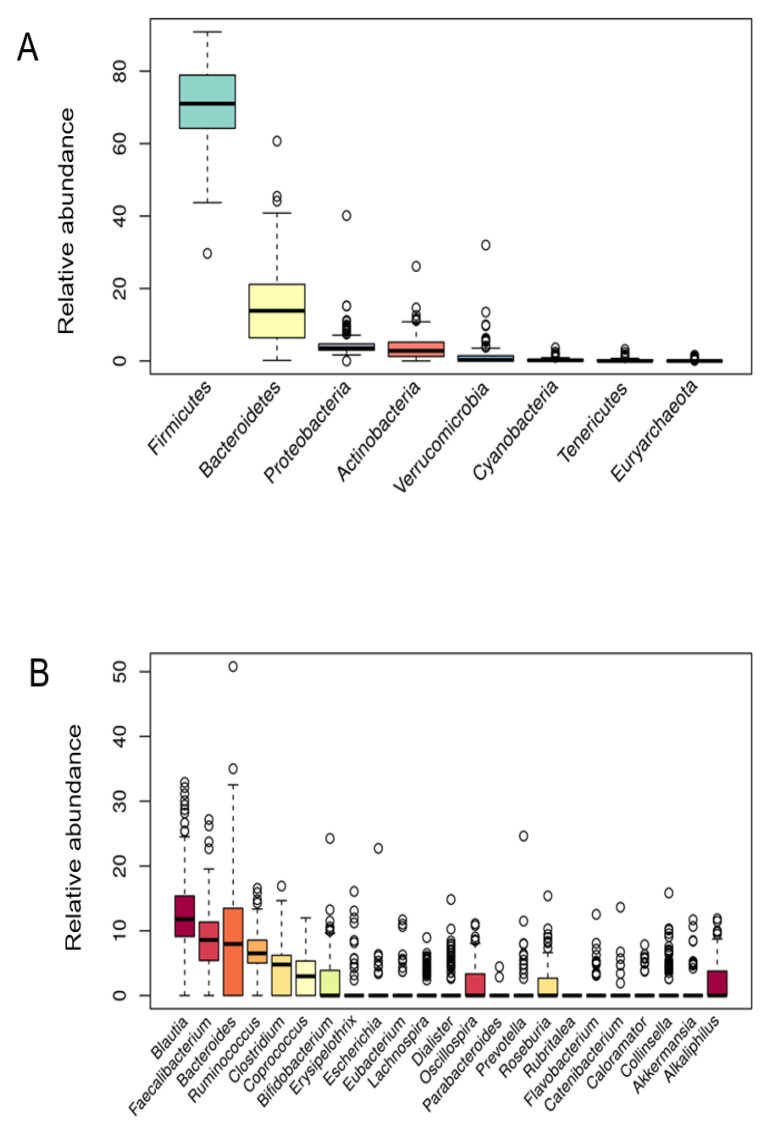
Bacterial phyla (**A**) and genera (**B**) relative abundance.

To characterize the bacterial microbiota composition, 16S rRNA gene sequencing on collected fecal samples was performed. In the present cohort study, the microbial profiles of 200 individuals were analyzed. The abundances of 8 bacterial phyla (Figure 1A) and 23 genera (Figure 1B) that were found in more than 10% of study participants are presented in Figure 1. Firmicutes, Bacterioidetes, and Proteobacteria phyla were detected in the microbiota of all participants. Firmicutes was the predominant group at the phylum level (71.02 ± 11.45) followed by Bacteroidetes (13.85 ± 10.20), Proteobacteria (3.52 ± 3.33), Actinobacteria (2.80 ± 3.25), and Verrucomicrobia (0.28 ± 2.87). Moreover, Firmicutes was the most dominant phylum in the vast majority of individuals (98%). Regarding taxonomic classification, the most abundant genera also belonged to Firmicutes phyla (*Blautia*, *Faecalibacterium*, *Ruminococcus*, and *Clostridium*). In addition to this, most of the bacterial population belonged to the *Blautia* genus (11.79 ± 5.84), followed by *Faecalibacterium* (8.59 ± 5.09), *Bacteroides* (7.97 ± 8.05), *Ruminococcus* (6.51 ± 3.17), and *Clostridium* (4.79 ± 3.48).

### 3.3. Microbiota Cluster Analysis

**Figure 2:**
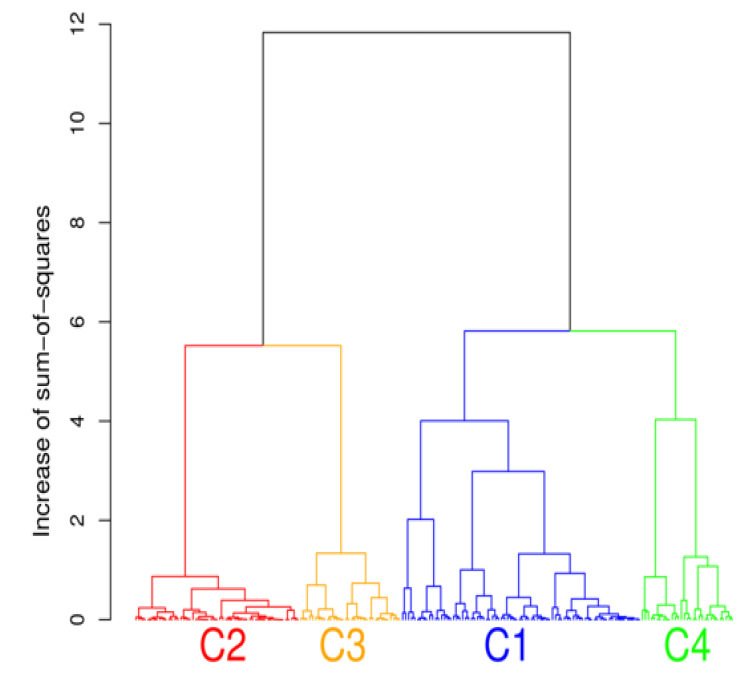
The cluster analysis showing the presence of four distinct community types of gut bacteria phyla.

Hierarchical clustering was performed to analyze the microbiota phyla data set. In the tradeoff between having a higher number of clusters, where better focus is achieved but clusters can contain too few variables, and a lower number of clusters with excessive variations, four clusters were selected. Choosing four clusters also resulted in interpretable results. The numbers of members (study participants) in each cluster were 80, 55, 34, and 31 for C1, C2, C3, and C4, respectively. The hierarchical clustering of microbiota data is presented by a tree diagram (dendrogram) in Figure 2.

#### 3.3.1. Microbial Abundance over Clusters

**Figure 3:**
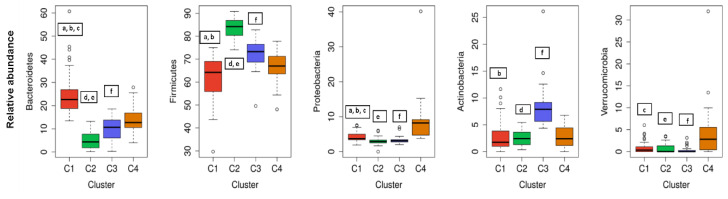
The cluster distribution of five most abundant bacterial phyla. Relative abundance per cluster of the five most abundant bacteria phyla present in our cohort. Each box shows the median plus interquartile range of the relative abundance of bacterial phyla. Colors show the cluster. Statistical significance differences (*p* < 0.05) between clusters in each phylum are presented with symbols a, b, c, d, e, and f: a, statistical significance (*p* < 0.05) between C1 and C2. b, statistical significance (*p* < 0.05) between C1 and C3. c, statistical significance (*p* < 0.05) between C1 and C4. d, statistical significance (*p* < 0.05) between C2 and C3. e, statistical significance (*p* < 0.05) between C2 and C4. f, statistical significance (*p* < 0.05) between C3 and C4.

**Figure 4:**
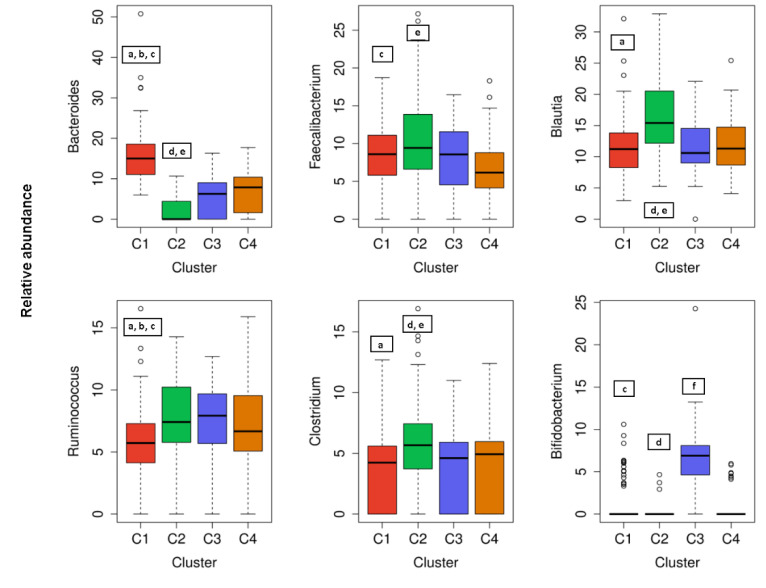
The cluster distribution of six most abundant bacterial and genera. Relative abundance per cluster of the six most abundant bacteria genera present in our cohort. Each box shows the median plus interquartile range of the relative abundance of bacterial genera. Colors show the cluster. Statistical significance differences (*p* < 0.05) between clusters in each genus are presented with symbols a, b, c, d, e, and f: a, statistical significance (*p* < 0.05) between C1 and C2. b, statistical significance (*p* < 0.05) between C1 and C3. c, statistical significance (*p* < 0.05) between C1 and C4. d, statistical significance (*p* < 0.05) between C2 and C3. e, statistical significance (*p* < 0.05) between C2 and C4. f, statistical significance (*p* < 0.05) between C3 and C4.

The cluster analysis suggested the presence of four distinct community types (Figure 2). All statistically significant differences between clusters regarding bacterial phyla and genera are presented in Figure 3; Figure 4. Based on the cluster distribution of phyla (Figure 3), the highest abundance of Firmicutes in the C2 cluster was observed, followed by C3, C4, and C1. Moreover, the C2 cluster has been characterized by a significantly lower abundance of Bacterioidetes, Proteobacteria, and Verrucomicrobia phyla. Among all, the C1 cluster was characterized by the highest proportion of Bacteroidetes and at the same time by the lowest proportion of Firmicutes and Actinobacteria phyla. The C4 cluster has been characterized by the highest proportion of Proteobacteria and Verrucomicrobia. Furthermore, the abundance of Actinobacteria phyla was significantly higher in C3, followed by C2, C4, and C1. Additionally, the C3 cluster was characterized also by a significantly higher proportion of the *Bifidobacterium* genus (Figure 4) when compared to other clusters C1 (*p* = 0.041), C2 (*p* = 0.010), and C4 (*p* = 0.027).

Regarding the most abundant genera of participants gut microbiota, a significantly (*p* < 0.05) higher proportion of *Clostridium* and *Blautia* was observed in C2 than in the C1, C3, and C4 clusters (Figure 4). In addition, a significantly (*p* < 0.05) higher proportion of *Bacteroides*, and a significantly lower proportion of *Ruminococcus* was observed in C1 than in the C2, C3, and C4 clusters (Figure 4). When considering less abundant genera of gut bacteria, there was also a statistically significant higher abundance of the genera as follows: *Prevotella* in the C1 cluster compared to C3 or C2; *Cyanobacteria* in the C1 cluster compared to C2 or C4 and *Erysipelothrix* in the C4 cluster compared to C1, C2, or C3 (data not shown). Nevertheless, a dominant genus can be assigned to an individual cluster as follows: *Bacteroides* to C1, *Blautia* and *Clostridium* to C2, *Bifidobacterium* to C3, and *Erysipelothrix* to the C4 cluster.

#### 3.3.2. The Most Important Variables for Classifying Subjects into Clusters

Table 2 lists the top 15 ranked representative variables for each cluster according to the relative model measure of variable importance. The most important variables (top five) for classifying subjects into clusters were measures of obesity (WHR, BMI, and visceral fat index), blood pressure, total body water, energy intake, total fat and olive oil intake, and total fiber intake and total water intake. Total fat intake was shown as the most important variable for classifying subjects into cluster 3; indeed, a score of importance was 91.0 for C3, 68.7 for C2, 64.0 for C1, and 60.5 for C4. The most important predictor with the highest score of importance (88.2) of the C2 cluster was BMI, a known measure of obesity. The BMI was also the second most important predictor for C1 (81.2) and the third for C3 (81.3). Furthermore, total fiber intake gained the highest score of importance (86.1) for classifying subjects into C1 and was the second most important for the C4 cluster. In addition, water intake with the highest score of importance (100) was the most important predictor for classifying subjects into C4. Among the 15 most important variables of the present prediction model were also sleeping hours, age, gender, metabolic age, EER, MEDAS, and dietary intake as follows: vitamins A, B9, and E; protein; saturated fatty acids; monounsaturated fatty acids; olive oil; vegetable fiber; carbohydrate; and milk and milk products intake, respectively (Table 2).

Moreover, for most of the highly ranged cluster predictors, there were statistically significant differences between clusters (Table 3). According to the most important parameters, there were significant differences in anthropometric parameters between clusters, whereas participants in C2 had significantly higher BMI, WHR, blood pressure, and visceral fat index, especially in comparison to participants in C1 (Table 3). The majority of the participants in C2 were overweight (BMI between 25 to 29.9 kg/m^2^), and 20% of the participants in C2 had two components of metabolic syndrome. When considering nutritional intake, the higher energy, fat, and saturated fat intake but lower carbohydrates intake were significant for C2 in comparison to subjects in other three clusters (Table 3). However, there were no statistically significant differences in total fiber or olive oil intake between the subjects in C1 and C4, and it was higher for both when compared to subjects in C2 and C3. On the other hand, subjects in C3 had significantly lower fat and saturated fat intake and higher carbohydrates intake in comparison to other clusters. Moreover, subjects in C3 had the highest consumption of milk and milk products (Table 3). Regarding gender, the majority of females can be found in the C1 cluster, whereas C2 was representative for males. The C2 cluster was also represented by the lowest number of average sleeping hours (7.5 ± 0.7). Nevertheless, the youngest participants (32.6 ± 6.1) were clustered in C3.

## 4. Discussion

Gut microbiota is a diverse consortium of microorganisms that can remarkably influence the wellbeing of the host. Although the adult-like microbiota establishes already in the first six years of a children’s life [30], composition and diversity can be affected by many factors further in life. The studies investigating various demographic, environmental, clinical, and genetic factors in correlation to microbial community diversity, composition, and/or function showed promising results. However, most of the variables were investigated individually not taking into consideration the collateral role of other individual-specific variables that often leads to inconsistent results [20]. Therefore, in this cross-sectional study, an extensive data set of 200 healthy Slovenian individuals was obtained with the aim of elucidating the variables that could predict the allocation of the subjects into clusters according to their gut microbiota composition. Regarding the distribution of major bacterial phyla and genera of healthy Slovene individuals in this study, it was comparable with previous population-based core microbiome studies [17,20,31]. Our data were consistent with Firmicutes and Bacteroidetes phyla covering the vast majority of the dominant human gut bacteria [32] with the relative abundance of Firmicutes 71.02 ± 11.45%, followed by 13.85 ± 10.20% of Bacteroidetes (Figure 1). In the hierarchical clustering of gut microbiota data set, five bacteria phyla with the highest abundance were included. The optimal number of clusters was set by the value four among which there were statistically significant differences in bacterial phyla abundance, and each cluster had a group representative (Figure 3). Namely, Bacteroidetes phylum was the most prevalent in the C1 cluster, Firmicutes in C2, Actinobacteria in C3, and in the C4 cluster Proteobacteria and Verrucomicrobia phyla. As expected, four (4/5) of the most abundant bacteria genera belonged to the Firmicutes, the dominant phylum in the vast majority of individuals (98%). However, representative genera of the individual clusters were *Bacteroides* and *Prevotella* in C1, *Blautia* and *Clostridium* in C2, *Bifidobacterium* in C3, and *Erysipelothrix* in the C4 cluster (Figure 4).

Several non-genetic variables, including demographic, lifestyle and environmental factors were associated with fecal microbiome diversity in healthy individuals [20]. Regarding the relative index as a measure of the variable importance in our SVM model, obesity measures (BMI, WHR, and visceral fat index), blood pressure, total body water, and dietary variables (energy intake, total fat and olive oil intake, total fiber intake, and water intake) were among first five in classifying subjects into clusters (Table 2). A lower abundance of Bacteroidetes with a proportional increase of bacteria belonging to Firmicutes phylum was observed in individuals with obesity [33], confirming our model since all three measures of obesity are significantly higher for subjects in the C2 cluster. Moreover, the differences in *Firmicutes*/*Bacteroidetes* ratio, which has great importance in the development of obesity, was shown to be influenced by the grade of obesity [34]. It is clear that our model identified several previously reviewed variables of gut microbiota, including blood pressure. Although only asymptomatic individuals were included in our study, subjects in the C2 cluster had higher blood pressure in comparison to subjects in other clusters (Table 3). In addition, 20% of the subjects in the C2 had two components of the metabolic syndrome (large waist circumference and increased blood pressure). However, only two out of five criteria for metabolic syndrome were measured in this study; fasting glucose and lipids were not assessed. Therefore, it was not possible to determine how many patients met the criteria of metabolic syndrome. Nevertheless, C2 was characterized by a significantly higher *Firmicutes/Bacteroidetes* (F/B) ratio when compared to other clusters, supporting previous results, e.g., for hypertensive individuals [35]. Moreover, high blood pressure was associated with gut microbiota dysbiosis, both in animal and human hypertension [36].

It is clear that the composition of the human gut microbiota fluctuates in response to the nutritional composition of the diet through life [37,38]. Due to its geographical location, the dietary pattern of most Slovenian people follows the guidelines of the Mediterranean diet. Accordingly, our result showed that most of the participants (59%) had moderate or even high (11%) adherence to the Mediterranean diet. Such dietary pattern consists of a high variety of vegetables and fruits, legumes and whole grains, olive oil as a main source of fat and is coupled with moderate consumption of red wine [39]. Fiber, mono- and poly-unsaturated fatty acids, antioxidants, and polyphenols rich Mediterranean diet has demonstrated beneficial modulation of the gut microbiota in humans as well as in experimental animal models [40]. The last could also be observed on the basis of this study results since the assignment of subjects in clusters is highly predictable by the consumption of dietary parameters characterizing the Mediterranean diet (Table 2). MUFAs such as the oleic acid present in extra virgin olive oil are among the main components of the “Mediterranean diet”. A recent systematic review [41] showed that high-MUFA diets have no effect on richness/diversity indexes, phylum distribution, or *Bacteroidetes/Firmicutes* ratio. However, at family and genera level, MUFA-rich diets could be positively correlated with *Parabacteroides*, *Prevotella*, and *Turicibacter* genera and *Enterobacteriaceae* family, and with a lower number of *Bifidobacterium* genus. In our model, olive oil intake was one of the most important predictors for classifying subjects in C1 and C4, where subjects had significantly higher olive oil intake and also a higher abundance of *Prevotella*.

By changing eating habits, the alternations in gut microbiota can be observed already after a single day, while the animal-based diet was showed to have a greater impact than the plant-based diet [16]. Correlations between dietary consumption parameters (e.g., fried products, raw fruits, and fish) and gut microbiome have previously been shown in the *Milieu intérieur* (MI) cohort—a population-based study of 1000 healthy individuals of western European ancestry, evenly stratified by sex and age [42]. Among dietary consumption parameters of the present study, total fat intake was the most important variable for classifying subjects into C3 with a score of importance 91.0, followed by 68.7 for C2, 64.0 for C1, and 60.5 as the C4 cluster predictor. Moreover, several studies described a decrease in Bacteroidetes and an increase in *Firmicutes* and *Proteobacteria* phyla in response to high-fat diet (HFD), specifically saturated fatty acids (SFAs) consumption [43]. In accordance, a higher abundance of Firmicutes was observed in C2, where subjects had statistically significant higher (*p* < 0.05) fat and saturated fat intake when compared to C1, C4, or the C3 cluster (Table 3). Accordingly, there was also a higher proportion of *Blautia* in C2 as it had already been shown before that high intake of SFAs was positively associated with the abundance of that genus [44]. In accordance with the present results, where a higher abundance of *Bifidobacterium* and *Bacteroides* and lower fat intake in C1 and C3 was observed, the same pattern was recognized by a recent systematic review [41] in the case of low fat/high carbohydrate diets in adults at increased risk of metabolic syndrome. The higher intake of total energy and a higher amount of carbohydrates in the diet has also been associated with lower gut microbiota diversity [12]. Furthermore, the growth and activity of preferred bacterial strains that can confer a beneficial physiologic effect on the host can be selectively stimulated by the dietary fiber intake [45,46]. Regarding total fiber intake in this study, subjects in the C1 and C4 cluster were characterized by higher values of fiber intake that was statistically significantly different when compared to C2 or the C3 cluster subjects. Correspondingly, the dominance of Bacteroidetes phylum in C1 was in accordance with previous studies showing an association of fiber and plant-derived polysaccharide-rich diet in children and adults to gut microbiota enriched in Bacteroidetes [16,47].

The C1 was characterized also by a higher abundance of *Prevotella* compared to C3 and C2, as shown before there was a positive correlation in fiber intake and this bacterial genus levels [48] and reduced during consumption of the animal-based diet [16]. Moreover, the C1 cluster was characterized by the lowest proportion of the Actinobacteria phylum, including low levels of *Bifidobacterium* genus, also common for populations in which meat and/or dairy consumption is low to absent, such as vegans [49]. On the other hand, the consumption of milk and milk products was significantly higher for subjects in the C3 cluster, which correlates to the highest abundance of *Bifidobacterium* among clusters. Dairy product consumption has widely been explored and was shown to increase the presence of potentially beneficial bacteria, particularly, *Bifidobacterium* genus [50]. Among the first five variables with the strongest prediction power, the water intake was also listed and was significantly higher for subjects in C1 and C4 compared to C3 (Table 3). As shown before, quantitative or qualitative changes in habitual water drinking habits can affect the abundance of several bacterial genera, whereas the abundance of Bacteroides could be assigned to higher water consumption [51]. Surprisingly, alcohol consumption and tobacco consumption were not among top-ranked variables. However, this study’s subjects were healthy individuals with the mean age of 35.4 ± 7.0 years, and in principle, most of them were not regular smokers or severe alcohol consumers. Nevertheless, the smokers of the present study clustered almost equally in all clusters (data not shown). Such behaviors or health status are however most often linked to a deviation of gut microbiota composition compared to healthy individuals [52].

For mammalian hosts, vitamins are essential micronutrients obtained from the diet or through the metabolism of commensal GI bacteria. In the present study, vitamins, A, B9, and E intake were found among the first 15 strongest predicting variables of cluster classification (Table 2). All of them were found to be crucial for the classification of subjects into the C3 cluster. Accordingly, in the C3 cluster, represented by a higher abundance of *Bifidobacterium* genus, subjects with a significantly lower vitamin A intake were classified. As shown by Liu et al. (2017), vitamin A supplementation of children with autism spectrum disorder resulted in significantly decreased *Bifidobacterium* [53]. Especially for water-soluble vitamins (e.g., vitamin B family and vitamin C), it is important to consume a diet containing the necessary amounts of these vitamins. There was a significantly higher vitamin B9 intake for subjects in C1 and C3 compared to C2 or C4 (Table 3) supporting the research of bacterial richness, and composition differed significantly by the consumption of folate and B vitamin group [54].

Nevertheless, gender, age, and sleeping hours were also among the first 15 most important variables for classification; gender for C1, C2, and C3 clusters but not for C4; age for C3 and C4 clusters; and sleeping hours for C3 cluster (Table 2). Both demographical variables were shown to be correlated not only to microbial composition and diversity but also to functional richness [12]. The differences in gut microbiota between males and females, such as higher levels of *Bacteroides–Prevotella* group (Bacteroidetes phylum) in males [55] and a higher proportion of Firmicutes in females [34], were shown before. However, due to the inter-individual heterogeneity of subjects, the results of studies describing gender-related differences in gut microbiota have shown to be contradictory. In accordance with Haro et al. (2016), a global pattern of the bacterial community tested by NGS Illumina platform confirmed the lower abundance of Bacteroidetes in females as compared with males when BMI is around 25 kg/m^2^ [34,56]. However, in the present study a heterogeneous group of lean, overweight, and obese subjects were included, and in the C1 cluster where there was a greater abundance of Bacteroidetes phylum, there was also the highest proportion of females (Figure 3).

Age-related changes of human gut microbiota, characterized by a different abundance of bacteria in various age groups, have been shown [57,58,59]. Moreover, the multivariate unsupervised analysis on genera abundance profile revealed the continuous aging progression of human gut microbiota along with the host aging process [60]. Regardless of the narrow age group of our study, the youngest participants were located in the C3 cluster. The last was represented also by the highest proportion of Actinobacteria phyla, which is in accordance with a previous publication showing substantial decrease of Actinobacteria relative abundance with age [58]. Although the data on the relationship between sleep and gut-microbiome composition are scarce, a recent study found that total microbiome diversity was positively correlated with increased sleep efficiency and total sleep time [61]. As shown by Benedict et al. (2016), increased *Firmicutes:Bacteroidetes* ratio followed restricted sleep vs. normal sleep [62]. Based on our study results, the increased abundance of Firmicutes was significant for the C2 cluster where participants with the lower average sleeping hours were clustered.

According to other authors’ results [34,56], there are several factors that need to be taken into consideration simultaneously when looking for variables linked to gut microbiota composition. Therefore, our model shows a promising result while healthy individuals can be clustered on the basis of gut microbiota composition, thus indicating different strong predictor parameters including demographics, anthropometrics, lifestyle (especially dietary intake), and socio-economic status. As the most prevalent predictors of all described clusters, measures of obesity (BMI, WHR, and visceral fat index) and blood pressure were observed. As diet stands for one of the modifiable risk factors for many non-communicable diseases, there is a high level of evidence supporting the efficacy of dietary interventions for both influencing disease risk and improving disease outcomes. Nevertheless, there is still an open window for studying micronutrition intake on gut microbiota that could have a potential clinical/therapeutic implication in different non-communicable diseases.

## 5. Conclusions

The SVM model presented in this study was shown as a valuable tool to classify healthy individuals based on their gut microbiota composition. Moreover, classification can be performed by lifestyle parameters, such as measures of obesity and dietary consumption habits. Using a large data set, this model could help us to predict the gut microbiota composition of a healthy individual. However, there is a need for additional research work to investigate the accuracy of the presented model in predicting gut microbiota of the most abundant genera and phyla on a larger group of healthy individuals.

## Figures and Tables

**Table 1:** Basic characteristics of the participants included in the study (*n* = 200).

Characteristics	
**Age years (mean ± SD)**	35.4 ± 7.0
**Gender**	
Female	117 (58.5%)
Male	83 (41.5%)
**Level of education**	
Primary education	5 (2.5%)
Secondary education or high school	68 (34%)
University degree (Bachelor’s, Master’s, Doctor’s)	127 (63.5%)
**BMI kg/m^2^ (mean ± SD)**	24.2 ± 3.5
**Weight (kg) (mean ± SD)**	73.1 ± 15.4
**WHR = waist (cm)/hip (cm) (mean ± SD)**	0.87 ± 0.07
**Visceral fat index**	4.7 ± 2.9
**Blood pressure**	
Systolic	125.8 ± 13.2
Diastolic	81.3 ± 9.8
**Hours of sleep (mean ± SD)**	7.7 ± 0.8
**Smoker**	
Yes	54 (27%)
No	146 (73%)
**Alcohol consumption habits** (mean g/day ± SD)	14.5 ± 13.6
Up to 5 g/day	78 (39%)
5–12 g/day (W) and 5–20 g/day (M)	60 (30%)
>12 g/day (W) and >20 g/day (M)	62 (31%)
**Dietary intake**	
EER kcal (mean ± SD)	3093 ± 776
Energy intake (kcal/day)	1892 ± 1000
Total fat (%)	32.07 ± 9.96
Carbohydrate (%)	49.05 ± 10.18
Protein (%)	18.14 ± 6.48
Fiber (g/day)	19.37 ± 13.67
**MEDAS (mean ± SD)**	7.7 ± 2.4
0–6 points	60 (30%)
7–10 points	118 (59%)
11–14 points	22 (11%)
**Physical activity (kcal/week)**	4219 ± 335

Data are presented as mean values ± SD or N (%). Estimated Energy Requirements (EER); Mediterranean diet adherence (MEDAS).

**Table 2:** The list of demographical, social, nutritional, and medical predictors of four clusters by relative value of importance.

Clusters of Subjects								
C1			C2		C3		C4	
Predictor and Relative Value of Importance								
1	Total fiber intake	86.1	BMI	88.2	Fat intake	91.0	Water intake	100
2	BMI	81.2	Blood pressure	81.7	Water intake	90.2	Total fiber intake	86.1
3	Water intake	81.1	Total body water	77.7	BMI	81.3	Visceral fat index	77.7
4	Blood pressure	77.4	Waist/hip ratio	76.5	Energy intake	81.1	Energy intake	77.4
5	Energy intake	76.4	Visceral fat index	73.8	Visceral fat index	77.7	Olive oil intake	74.8
6	Olive oil intake	74.1	Fat intake	68.7	Vitamin A intake	76.7	Milk and milk products intake	74.4
7	Visceral fat index	73.5	Saturated fatty acids intake	68.7	Vitamin B9 intake	75.2	BMI	68.8
8	Waist/hip ratio	68.0	Waist/hip ratio	64.9	Sleeping hours	73.5	Carbohydrates intake	68.4
9	Milk and milk products intake	67.9	Gender	64.7	Age	68.0	Fat intake	67.9
10	Fat intake	64.0	Energy requirements	64.7	Gender	67.7	Age	60.5
11	Saturated fatty acids intake	63.3	Legume intake	63.5	Saturated fatty acids intake	64.3	MEDAS	57.7
12	Gender	62.1	Protein intake	60.8	Monounsaturated fatty acids intake	63.0	Monounsaturated fatty acids intake	57.7
13	Energy requirements	58.2	Vegetable fiber intake	60.2	MEDAS	62.1	Protein intake	57.3
14	Carbohydrates intake	57.7	Metabolic age	59.8	Vegetable fiber intake	60.3	Vegetable fiber intake	56.6
15	Age	57.7	Carbohydrates intake	59.3	Vitamin E intake	59.9	Energy requirements	56.2

**Table 3:** Differences in the top ten predictors (gender, obesity parameters, energy intake, physical activity, and nutrient intakes) between four clusters.

	Clusters			
Predictors	C1 (*n* = 80)	C2 (*n* = 55)	C3 (*n* = 34)	C4 (*n* = 31)
Gender: *n* (F/M); % (F/M)	54/26; 68/32 ^a,b^	21/34; 38/62 ^d,e^	19/15; 55/45 ^f^	22/9; 71/29
Age (years)	36.0 ± 7.4 ^b^	36.4 ± 6.2 ^d^	32.6 ± 6.1 ^f^	35.8 ± 6.2
Obesity parameters				
BMI (kg/m^2^)	23.1 ± 3.3 ^a,b,c^	25.5 ± 3.4 ^d,e^	24.7 ± 3.3	24.0 ± 3.3
BMI > 25 kg/m^2^: *n*; %	20; 20% ^a,b,c^	31; 56% ^d,e^	14; 41%	12; 39%
Waist to hip ratio	0.85 ± 0.08 ^a^	0.89 ± 0.06 ^e^	0.87 ± 0.07	0.85 ± 0.05
Visceral fat index	3.9 ± 2.8 ^a,b,c^	5.7 ± 2.9 ^d,e^	4.8 ± 2.7	4.6 ± 3.0
Total body water (%)	55.6 ± 4.1 ^c^	55.8 ± 5.8 ^e^	54.2 ± 6.1	52.5 ± 4.3
Blood pressure (mmHg)	122 ± 12/78 ± 9 ^a,c^	130 ± 13/84 ± 10 ^d^	125 ± 12/81 ± 9	128 ± 13/84 ± 9
Presence of two metabolic syndrome components: *n*; %	6; 7.5% ^a^	11; 20% ^d,e^	1; 2.9%	3; 9.7%
Nutrition				
Energy intake (kcal/day)	1700 ± 433	1720 ± 486	1590 ± 1165	1666 ± 614
Water intake (g)	1080 ± 435 ^b^	1029 ± 322 ^d^	840 ± 553 ^f^	1097 ± 615
Fat intake (%)	30.7 ± 9.6 ^a^	36.2 ± 10.1 ^d,e^	28.7 ± 9.3	31.8 ± 8.7
Saturated fat intake (%)	8.3 ± 4.6 ^a,b^	8.8 ± 4.8 ^d,e^	7.2 ± 4.0 ^f^	8.3 ± 4.8
Carbohydrates intake (%)	50.0 ± 9.0	46.4 ± 11.4 ^d^	52.2 ± 10.4	48.2 ± 8.2
Total fiber intake (g/day)	21.0 ± 14.0 ^a,b^	17.0 ± 8.8 ^e^	16.4 ± 12.2 ^f^	22.1 ± 13.1
Vitamin A (µg/day)	1352 ± 1837 ^b^	1420 ± 1585 ^d^	960 ± 630	1243 ± 1178
Vitamin B9 (µg/day)	408 ± 249 ^a^	338 ± 283 ^d,e^	412 ± 222	374 ± 208
Olive oil intake (g/day)	26 ± 24 ^a,b^	19 ± 21 ^e^	18 ± 13 ^f^	28 ± 23
Milk and milk products	108 ± 101 ^a,b^	162 ± 149 ^e^	174 ± 129 ^f^	124 ± 119
Other parameters				
Sleeping hours (hours/day)	7.7 ± 0.7	7.5 ± 0.7 ^d^	8.0 ± 0.7	8.0 ± 0.9

Female (F); Men (M); BMI, body mass index; EER, Estimated Energy Requirement. ^a^ Statistical significance (*p* < 0.05) between C1 and C2. ^b^ Statistical significance (*p* < 0.05) between C1 and C3. ^c^ Statistical significance (*p* < 0.05) between C1 and C4. ^d^ Statistical significance (*p* < 0.05) between C2 and C3. ^e^ Statistical significance (*p* < 0.05) between C2 and C4. ^f^ Statistical significance (*p* < 0.05) between C3 and C4.

## Supplementary Materials

The following are available online at https://www.mdpi.com/2072-6643/12/9/2695/s1. Table S1: Data with relative abundances of phyla and genera of 200 subjects that were included in the present study. Table S2: Description of variables used for classification challenges in Support Vector Machine model.
