# Supplementary material for: Obesity Measures and Dietary Parameters as Predictors of Gut Microbiota Phyla in Healthy Individuals
Source: Nutrients. 2020 Sep 3;12(9):2695. doi: 10.3390/nu12092695 (PMC7551767; doi:10.3390/nu12092695)
Supplement: Supplementary file 1 [file nutrients-12-02695-s001.zip › nutrients-881593-supplementary Table S2 Description of variables used for classification challenges in Support Vector Machine model.docx]

**Table S2:** Description of variables used for classification challenges in Support Vector Machine model.

| **Parameter** | | **Description** |
| --- | --- | --- |
| **Subject’s basic characteristics and lifestyle parameters** | | |
| 1 | Age | Subject's age |
| 2 | Gender | Female or male |
| 3 | Marital status | Single, married, widowed, separated, divorced |
| 4 | Education | Subject's educational level |
| 5 | Working status | Subject’s working status (employed, unemployed, retired, student) |
| 6 | Income | Income sources |
| 7 | Capital | Total capital (in EUR) |
| 8 | Alcohol | Alcohol consumption (g/day) |
| 9 | Tobacco | Tobacco consumption (number of cigarettes per day) |
| 10 | PA | Physical activity (min/day) |
| 11 | EER | Estimated energy requirements (kcal/day) |
| 12 | Sleeping hours | Sleeping hours per day (h/day) |
| 13 | Meals | Number of meals per day |
| **Overall health status** | | |
| 14 | Systolic Blood Pressure | Systolic Blood Pressure (mm Hg) |
| 15 | Diastolic Blood Pressure | Diastolic Blood Pressure (mm Hg) |
| 16 | Overall Health | Subjective self-perception of physical health (score) |
| 17 | Emotional health | Subjective self-perception of emotional health (score) |
| 18 | Pain | Subjective self-perception of pain (score) |
| **Subject’s anthropometric characteristics** | | |
| 19 | BMI | Body mass index (kg/m^2^) |
| 20 | Muscle Mass | Muscle mass (%) |
| 21 | ICC | Waist to hip ratio |
| 22 | Total Body Water | Total body water (%) |
| 23 | Metabolic Age | Metabolic age (calculated from BIA, years) |
| 24 | Body Fat | Body fat (%) |
| 25 | Visceral Fat Index | Visceral fat index |
| **Macronutrients and micronutrients intakes** | | |
| 26 | EI | Total energy intake (kcal) |
| 27 | Water intake | Water intake (g/day) |
| 28 | CHO | Total carbohydrate intake (% of energy intake) |
| 29 | GL | Glycemic load index |
| 30 | P | Total protein intake (% of energy intake) |
| 31 | FAT | Total fat intake (% of energy intake) |
| 32 | SAF | Total saturated fat intake (% of energy intake) |
| 33 | MUFA | Total monounsaturated fat intake (% of energy intake) |
| 34 | PUFA | Total polyunsaturated fat intake (% of energy intake) |
| 35 | FIBER | Total fiber intake (g/day) |
| 36 | Veg fiber | Vegetable fiber intake (g/day) |
| 37 | Fruit fiber | Fruit fiber intake (g/day) |
| 38 | Legumes fiber | Legumes fiber intake (g/day) |
| 39 | VitA | Vitamin A intake (µg/day) |
| 40 | VitD | Vitamin D intake (µg/day) |
| 41 | VitE | Vitamin E intake (mg/day) |
| 42 | VitK | Vitamin K intake (µg/day) |
| 43 | VitC | Vitamin C intake (µg/day) |
| 44 | VitB1 | Vitamin B1 intake (mg/day) |
| 45 | VitB2 | Vitamin B2 intake (mg/day) |
| 46 | VitB3 | Vitamin B3 intake (mg/day) |
| 47 | VitB6 | Vitamin B6 intake (mg/day) |
| 48 | VitB9 | Vitamin B9 intake (µg/day) |
| 49 | VitB12 | Vitamin B12 intake (µg/day) |
| 50 | Ca | Calcium intake (mg/day) |
| 51 | Fe | Iron intake (mg/day) |
| 52 | Na | Sodium intake (mg/day) |
| 53 | Mg | Magnesium intake (mg/day) |
| 54 | K | Potassium intake (mg/day) |
| 55 | I | Iodine intake (mg/day) |
| 56 | Se | Selenium intake (mg/day) |
| 57 | Zn | Zinc intake (mg/day) |
| 58 | Cr | Chromium intake (mg/day) |
| 59 | P | Phosphates intake (mg/day) |
| **Food groups** | | |
| 60 | Vegetable | Vegetable (unit/day) |
| 61 | Fruit | Fruit (unit/day) |
| 62 | Legumes | Legumes (unit/day) |
| 63 | Starchy food | Starchy food (unit/day) |
| 64 | Sugars | Starchy food (unit/day) |
| 65 | Milk and milk products | Milk and milk products (unit/day) |
| 67 | Meat and meat products | Meat and meat products (unit/day) |
| 68 | Fat and fatty food | Fat and fatty food (unit/day) |
| **Adherence to Mediterranean diet** | | |
| 69 | MEDAS | Adherence to Mediterranean diet (total MEDAS score) |
| 70 | Olive oil | Olive oil intake (g/day) |
